# Supplementary material for: OpenMM 7: Rapid development of high performance algorithms for molecular dynamics
Source: PLoS Comput Biol. 2017 Jul 26;13(7):e1005659. doi: 10.1371/journal.pcbi.1005659 (PMC5549999; doi:10.1371/journal.pcbi.1005659)
Supplement: S1 Source Code — (ZIP) [file pcbi.1005659.s002.zip › OpenMM-7.0.1-Source/docs/api-c++/generated/OpenMM.VirtualSite.html]

VirtualSite — OpenMM @OPENMM\_MAJOR\_VERSION@.@OPENMM\_MINOR\_VERSION@ documentation


# VirtualSite¶

*class* `OpenMM::``VirtualSite`¶
:   A `VirtualSite` describes the rules for computing a particle’s position based on other particles. This is an abstract class. Subclasses define particular rules. To define a virtual site, create an instance of a `VirtualSite` subclass and then call setVirtualSite() on the `System`.

    Methods

    |  |  |
    | --- | --- |
    | `~VirtualSite` |  |
    | `getNumParticles` | Get the number of particles this virtual site depends on. |
    | `getParticle` | Get the index of a particle this virtual site depends on. |

    `~VirtualSite`()¶

    int `getNumParticles`() *const*¶
    :   Get the number of particles this virtual site depends on.

    int `getParticle`(int *particle*) *const*¶
    :   Get the index of a particle this virtual site depends on.

        |  |  |
        | --- | --- |
        | Parameters: |  |

        - **particle** – the particle to get (between 0 and `getNumParticles()`)

        |  |  |
        | --- | --- |
        | Returns: | the index of the particle in the `System` |

High performance molecular simulation on GPUs


### Navigation

- Core Objects
- Forces
- Integrators
- Extras

©2015, Stanford University and the Authors.
|
Powered by Sphinx 1.3.1
& Alabaster 0.7.7
|
Page source
